# Integration of color, orientation, and size functional domains in the ventral pathway

**DOI:** 10.1117/1.NPh.4.3.031216

**Published:** 2017-05-27

**Authors:** Geoffrey M. Ghose, Daniel Y. Ts’o

**Affiliations:** a University of Minnesota, Department of Neuroscience, Center for Magnetic Resonance Research, Minneapolis, Minnesota, United States; b SUNY Upstate Medical University, Department of Neurosurgery, Syracuse, New York, United States

**Keywords:** functional organization, extrastriate visual cortex, optical imaging, areal specialization, color and form vision

## Abstract

Functional specialization within the extrastriate areas of the ventral pathway associated with visual form analysis is poorly understood. Studies comparing the functional selectivities of neurons within the early visual areas have found that there are more similar than different between the areas. We simultaneously imaged visually evoked activation over regions of V2 and V4 and parametrically varied three visual attributes for which selectivity exists in both areas: color, orientation, and size. We found that color selective regions were observed in both areas and were of similar size and spatial distribution. However, two major areal distinctions were observed: V4 contained a greater number and diversity of color-specific regions than V2 and exhibited a higher degree of overlap between domains for different functional attributes. In V2, size and color regions were largely segregated from orientation domains, whereas in V4 both color and size regions overlapped considerably with orientation regions. Our results suggest that higher-order composite selectivities in the extrastriate cortex may arise organically from the interactions afforded by an overlap of functional domains for lower order selectivities.

## 1 Introduction

Much of our understanding of how the cerebral cortex organizes and processes information is based on observations regarding the functional organization of the primate cortical areas associated with vision. At the scale of hundreds of microns, there is strong anatomical and physiological evidence for functional organization within striate cortex (V1) in which nearby neurons tend to exhibit matching preferences for ocular input, orientation, and color.^1^^–^^8^ At the scale of centimeters, each visual area has a complete representation of the visual hemifield. Earlier lesion and electrophysiological studies of the extrastriate visual areas led to the hypothesis that each of these visual areas is specialized for the analysis of distinct visual attributes.^9^^,^^10^ These studies further suggested that extrastriate areas could be grouped into two functional streams: a dorsal stream-containing area MT devoted to position and motion analysis, and a ventral stream-containing area V4 that is associated with form and object processing.^11^^,^^12^

However, clear functional differences, both within and between areas of the ventral visual stream, have been difficult to demonstrate.^13^ For example, while early reports suggested that area V4 was specialized for color processing,^14^^–^^16^ most systematic electrophysiological surveys of cells within areas V2 and V4 have reported similar incidences of color and orientation selectivities between the two areas.^17^^–^^19^ This is consistent with lesion studies of V4, which have reported modest effects on color perception.^20^^,^^21^ This situation is potentially analogous to the relationship between V1 and V2. Although single cell surveys have revealed clear differences between the areas, such as the lack of monocular cells within V2 and an increase in receptive field size in V2, they also have demonstrated that both V1 and V2 contain cells that are similarly selective for attributes, such as orientation, color, and spatial frequency. The two areas differ significantly, however, with respect to the spatial organization of these attributes along the cortical surface. This suggests that certain visual areas may be better distinguished not by the presence or absence of sensitivity to particular attributes but rather by how sensitivities are spatially organized. The degradation of retinotopic organization as one progresses from V1 to V2 to V4 supports this hypothesis. However, a more thorough test of the hypothesis clearly requires a comparison of how a variety of functional attributes are spatially organized in different areas.

One problem in testing this hypothesis is that functional organization within ventral extrastriate areas has proved difficult to study. Although classical electrophysiological techniques have suggested functional clustering in V2,^22^^–^^24^ V4,^18^^,^^25^^,^^26^ and inferotemporal cortex,^27^^,^^28^ ultimately these techniques are not well suited for a detailed study of functional organization because of several limitations. The first limitation is the necessary choice of specific stimulus sets that might preclude seeing differences. For example, if drifting bars are used to study single cells in areas V4 and MT, the areas appear to be quite similar.^29^ On the other hand, differences in stimulation can also make areas appear distinct in their selectivities. For example, the relatively large incidence of color selectivity among V4 cells reported in early studies^14^^–^^16^ appears to highly dependent on the methodology by which color properties were evaluated.^30^ The second is that, in order to maximize responses, different sets of stimuli are often used for each cell, complicating direct comparisons in the absence of strict stimulus separability. Although this can be avoided by using a standardized set of stimuli for every cell studied,^19^^,^^31^ such an approach is costly in terms of time and therefore, limits the sample size. A related concern is the diversity and sophistication of selectivities found. For example, in ventral area V4, investigators have found many selectivities including color, responses to non-Cartesian stimuli,^31^ depth,^32^ size,^33^ and object primitives.^34^^,^^35^ Single unit studies are limited by spatial sampling biases,^26^ particularly in regions with demonstrable functional organization. Finally, because neurons are examined sequentially rather than simultaneously, changes in anesthesia or behavioral state over time can affect comparisons between neurons.

Optical imaging solves some of these problems for areas that are on the cortical surface by allowing the simultaneous measurement of responses over a large population of neurons using large stimulus sets with high spatial resolution. In a previous report, we demonstrated the value of this approach by using optical imaging to reveal, for the first time, functional organization within area V4.^36^ We found organization for both orientation and size selectivities in V4. In the current study, we sought to extend these results by examining functional organization with respect to color and by quantitatively comparing the organization of color, size, and orientation selectivities in areas V2 and V4.

Our first goal was to identify whether chromatic-specific patches exist in areas V2 and V4. While previous optical imaging studies have revealed activations for specific color combinations,^37^ there has been no systematical sampling across color space for the two areas. Moreover, when color has been varied, it was varied in a perceptual space according to hue, rather than the physiological space that defines color representations in the early visual pathway.^38^ We find, when sampling across such a color space using both optical imaging and targeted electrophysiological recordings, chromatically specific regions of activation in both V2 and V4, which spanned the complete space of colors. Our second goal was to compare organization in V2 and V4. Although color-specific domains were similar in size in the two areas, isoluminant color regions were more numerous in V4 than in V2. Our final goal was to study the relationship between these different functional systems in the two areas. While in V2, color and especially size domains were largely segregated from orientation selective domains, no such segregation was visible in V4. These results support the hypothesis that even areas that appear similar by virtue of their selectivities to low-level visual attributes can be distinguished by the spatial organization of these selectivities. We propose that the increasing level of overlap between different primitive functional domains allows for the organization of more complicated selectivities in higher visual areas.

## 2 Methods

### 2.1 Surgical Preparation

Three *Macaca fasicularis* (2.5 to 5.1 kg) were used in these studies. Each animal was used in multiple (up to 4) imaging and electrophysiology sessions. All the experimental procedures were approved by the Institutional Animal Care and Use Committee of Baylor College of Medicine.

After initial sedation with ketamine hydrochloride (10  mg/kg), the animals were intubated endotrachially and a catheter was inserted in the saphenous vein. Animals were then anesthetized by continuous infusion of sufentanil (3 to 8  μg/kg-h). The level of anesthesia was continually monitored using an electrocardiogram and electroencephalogram. Once anesthetized, the animals were paralyzed with vercuronium (100  μg/kg-h), and artificially respirated to maintain an expired ${\mathrm{CO}}_{2}$ of 4%. A heating blanket was used to maintain rectal temperature at 38°C.

A craniotomy $∼1\;{\mathrm{cm}}^{2}$ was made over the lunate sulcus. A cylindrical chamber was then mounted on the skull surrounding the craniotomy with dental cement and sealed with bone wax. After pupil dilation and mydriasis (atropine 1%), the eyes were fitted with contact lenses of appropriate refraction so as to maintain focus on a CRT (Barco) placed 72 cm in front of the animal. Foveal positions were plotted with a fundus camera (Topcon). A small durotomy (∼1  mm) was made just posterior to the sulcus (V2) and anterior to the sulcus (V4). A layer of warm agar was applied to stabilize pulsations and a microelectrode was introduced into the two exposed cortical regions. Receptive field positions and sizes were plotted using multiunit and single unit activity. The eyes were then converged using a Risley prism over one eye to align the monocular receptive fields found in V2 to the same point on the monitor. After plotting receptive field locations for both the V2 and V4 regions, the entire dura was reflected and a sheet of transparent artificial dura (Tecoflex $∼1.5\;{\mathrm{cm}}^{2}$) was tucked under the dura in order to protect the cortex. The chamber was then filled with silicone oil and sealed to reduce cortical pulsations during imaging.

### 2.2 Optical Imaging and Visual Stimuli

Images of the cortical surface were acquired by a charge-coupled device camera (Photometrics) fitted with a macroscope lens assembly to reduce the depth of focus (∼300  μm). The cortical surface was uniformly illuminated by adjusting the position of optic fiber light guides. Green light (570 nm) was used to highlight surface vasculature and make focusing adjustments. Red light (630 nm) was then used to image small hemodynamic reflectance changes associated with neural activity while visual stimuli were presented on the CRT. During each stimulus presentation of about 2 s, 10 successive frames (192×144  pixels, 12  bits/pixel) were acquired, at 2 to 3 Hz. Depending on the lenses used, the spatial resolution of the images was 43 to 71  μm/pixel.

**Fig. 1:**
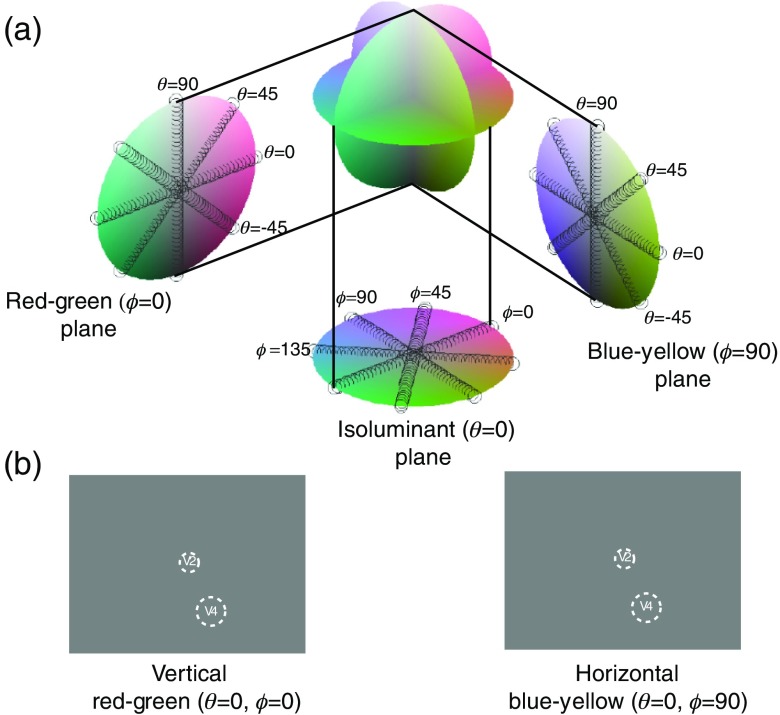
Stimuli used in optical imaging and single unit recording. (a) The DKL color space is described by three axes: two axes (red-green and blue-yellow) in which variations are isoluminant and cone-specific, and a third axis (Z) in which luminance varies without chromatic change. The origin is termed the equal-energy adaptation point: it corresponds with achromatic half-maximal luminance. We used stimuli that were sinusoidally modulated in space along nine different lines within this space. Described in spherical coordinates, these lines can be grouped according to their common angles into three different planes. For example, in the θ=0  deg plane, all points are isoluminant with the background but vary in their relative activation of cones. In this plane, our stimulus set included patches modulated along ϕ=0  deg, ϕ=45  deg, ϕ=90  deg, and ϕ=135  deg lines. Stimuli in the ϕ=0  deg (red-green) plane vary in luminance but do not differentially activate short-wavelength-sensitive (b) cones; stimuli in the ϕ=90  deg (blue-yellow) plane vary in luminance but do not differentially activate middle (G) and long (R) wavelength cones. In these ϕ constant planes, the stimulus set included patches modulated along the θ=−45  deg, θ=0  deg, θ=45  deg, and θ=90  deg lines. The gray-dashed cylinders in the three planes indicate the nine axes in color space in which gratings were modulated. (b) An array of nine gratings was presented. The array was aligned so that one patch was located over a plotted V2-receptive field while another patch was located over a plotted V4-receptive field. The spatial configuration and the spatial and temporal frequencies were kept constant over all variations of orientation and color.

In order to study color selectivity, the luminances of the CRT’s red, green, and blue phosphors were linearized and their spectra were reduced to three points in the Boynton–McLeod chromaticity diagram.^39^ The chromaticity coordinates and the luminance of the visual stimuli were calibrated using a spectroradiometer (PR650; Photo Research, Syracuse, New York). This allowed the construction of a Derrington–Krauskopf–Lennie (DKL) color space,^38^ which we used to specify our stimuli [Fig. 1(a)]. This three dimensional color space, which has been employed to construct stimuli for the examination of color selectivity among single neurons in the LGN,^38^ V1,^40^ and V2,^23^ is defined by two psychophysically defined chromatic axes (red-green and blue-yellow) and one luminance (achromatic) axis. Cells in the parvocellular layers of the LGN are optimally driven by chromatic stimuli that vary along one of the two axes: neurons that receive opponent R and G cone input are maximally sensitive to variations along the red-green axis, and neurons that receive opponent B and R+G cone input are maximally sensitive to variations along the blue-yellow axis. The origin of this space represents half-maximal activation along the three axes and is called the adaptation or equal-energy point. Recent electrophysiological studies have verified that the color selectivity in the DKL and CIE spaces are highly correlated for most V4 neurons.^41^

Because V4 neurons are often strongly suppressed by large stimuli,^33^ we wanted to avoid using the full-screen stimulation that is typically used for studies of functional organization in V1 and V2. However, by restricting the stimulation to a small single patch, we would be limiting any activation to a relatively small portion of cortex because of the visuotopic mapping present in areas V2^42^ and V4.^43^ Our compromise was to present an array of nine patches, each of which was 2 deg or less across. This array was configured such that one patch was placed on the electrophysiologically plotted V4-receptive field while another patch was placed over the plotted V2-receptive field [Fig. 1(b)]. The patches were on a background of gray mean luminance (the adaptation point) that was always present. All gratings were spatially modulated with a sinusoid around this point in the color space.

An imaging session typically consisted of 16 repetitions (two blocks of eight) of 16 to 32 different stimuli. Stimuli varied according to orientation and color and were constant with respect to position, size, drift direction, spatial frequency, temporal frequency, and contrast. For example, in most experiments the stimulus set was composed of achromatic gratings at four different orientations presented both monocularly and binocularly and gratings modulated in eight other directions in the color space [Fig. 1(b)] at two different orientations presented binocularly, for a total of 28 conditions (4×3+8×2). Spatial frequencies were usually ∼0.5  cycles/deg and temporal frequencies of ∼4  Hz. Each session also contained a “blank” stimulus in which no gratings were present. Conditions were pseudorandomly interleaved with an interstimulus interval of about 3 s. Acquired images were monitored on-line to ensure the absence of artifacts, such as bubbles in the silicone oil or dramatic cortical pulsations.

Off-line image analysis was done using custom software. Functional maps were constructed by subtracting sets of images acquired during complementary stimulation. For example, in order to compute an ocular dominance map, left eye and right eye condition groups are constructed by summing all acquired images from all imaging sessions associated with left and right eye stimulation. Before any summing across stimuli, single condition analysis was done (see below) on each stimulus image to ensure the absence of obvious artifacts such as bubbles. A stimulation “cocktail” map is constructed by summing the images associated with all different stimuli. The final map is obtained by subtracting condition group images normalized by the cocktail map. In many cases, condition group images were band-pass filtered by a broad Gaussian spatial frequency filter (centered at 0.8  cycle/mm, σ=2.4  cycle/mm for 45  μm/pixel images) to minimize low-frequency artifacts due to differences in illumination and high-frequency noise. Typically, the largest reflectance differences seen in our data set using this analysis were $∼10^{-3}$ (Fig. 8). In order to convert these differences into a visible map, the distribution of differences was computed over all pixels within the cortical region of interest. The distribution was then scaled and clipped at 8%-tiles out from the median value. The resulting pixel values are then linearly mapped to grayscale values from 0 to 255. In our experience, this algorithm tends to produce visual maps of optimal dynamic range. Because our imaging region was over the lunate sulcus, all our images had vascular artifacts due to the large vessels lying along the sulcus. To exclude these vascular artifacts, a map of the surface vasculature obtained with green light was used to mask different images by excluding pixels associated with large blood vessels.

In many analyses, we were interested in the activation associated with a single condition. In order to construct single condition activation maps, the first acquired frame (baseline) of a condition group was subtracted from the average of the last four frames of that condition group. To construct functional maps summarizing a range of conditions, multiple single conditions maps were color coded and added vectorially. In most cases, the color coding incorporates both selectivity and responsiveness such that hue represented stimulus preference, saturation represented stimulus selectivity, and intensity corresponded with responsiveness across the condition groups. For example, for orientation maps constructed in this way, a bright saturated pixel of a particular color indicates an orientation selective response in the region of the pixel, whereas a bright unsaturated pixel indicates large and uniform responses to all orientations.

Functional maps obtained through optical imaging incorporate both geometrical data, in the form of the particular locations of regions with consistent selectivity, and activation data, in the form of the actual reflectance signal observed at each point. We sought to quantify these aspects separately using unfiltered single condition reflectance data that were normalized by the “cocktail” average. To study the geometry of responsive regions, a contour map was formed outlining regions with 70% or higher of the maximal response using the MATLAB^®^ contour routine. Regions near the edges of the map or near the lunate sulcus were excluded from further analysis, and regions within V2 and V4 and for each direction of color space were analyzed separately. Centroids were computed for each region. An average radius for each region was computed by averaging the distance from this centroid to each vertex. The circular symmetry of each region was quantified by dividing the standard deviation of the radii by the average radius. Thus, a perfectly symmetric polygon such as a square would have a normalized standard deviation of 0. Because of our limited imaging window, we were unable to observe periodicities in functional architecture that spanned several millimeters. To quantify the geometrical relationship between response regions, we therefore compared the positions of the centers of neighboring response regions. The distance and direction (±90  deg) of connecting lines between neighboring regions were used in interareal and interstimulus comparisons of overall response region geometry.

In order to quantify selectivity along a particular stimulus dimension, such as orientation, maps for different single conditions of that dimension were analyzed on a pixel by pixel basis by a measure analogous to the saturation computation of red, green, and blue intensities. For each pixel, the average (A), maximum (max), and minimum (min) activation was tallied across the different stimulus conditions. The selective activation (sa) for each pixel was then sa=max*(1−min/A). Selective activation distributions were computed separately for V2 and V4 pixels across different stimulus dimensions, including orientation and color space axes.

Single condition analysis was also used to compute population activation functions for particular stimuli over areas V2 and V4. As was the case for the geometry analysis, no bandpass filtering was applied to the reflectance data. Because this analysis quantifies activation rather than selectivity, single condition data were not cocktail normalized. A cumulative response distribution for each stimulus condition was computed by compiling the responses across all pixels within a region of interest. As stated before, pixels associated with large vascular artifacts were excluded. The response distribution relates the percentage of pixels with a response greater than or equal to a criterion with that criterion. This distribution’s peak amplitude corresponds with the maximum signal observed in the region and its width describes the breadth of activity over the region. The shape of the function reflects the selectivity seen in single cells: if cells tend to be narrowly tuned in color space, then the distribution of activity to any particular color stimulus will also be narrow. In order to quantify these parameters, the response distributions were fit with Gaussians by minimizing RMS error.

### 2.3 Electrophysiology

After the imaging sessions were completed, the chamber was gradually drained and the protective film over the cortex was replaced with agar to allow electrophysiological recording with microelectrodes (∼0.5  MΩ @ 1 kHz). Specific functional domains were targeted by aligning functional maps with the surface vascular images. Single units from the superficial layers (0 to 500  μm) were isolated with an amplitude-based window discriminator (BAK Electronics). Receptive fields were quantitatively studied using stimuli similar to those used in our imaging paradigm. For example, when measuring the color selectivity of a cell, drifting sinusoidally modulated gratings were used of the spatial and temporal frequency as was used in imaging. However, when recording from single cells, only a single grating patch was presented and its size and position were adjusted according to estimates of the receptive field size and position. Spike times (resolution of 0.1 ms) from at least eight repetitions of each stimulus were collected. Optimal response rates were similar to those reported in previous studies of anesthetized V4, usually between 10 and 20  spk/s.^30^^,^^33^

## 3 Results

### 3.1 Color-Specific Regions in V2 and V4

**Fig. 2:**
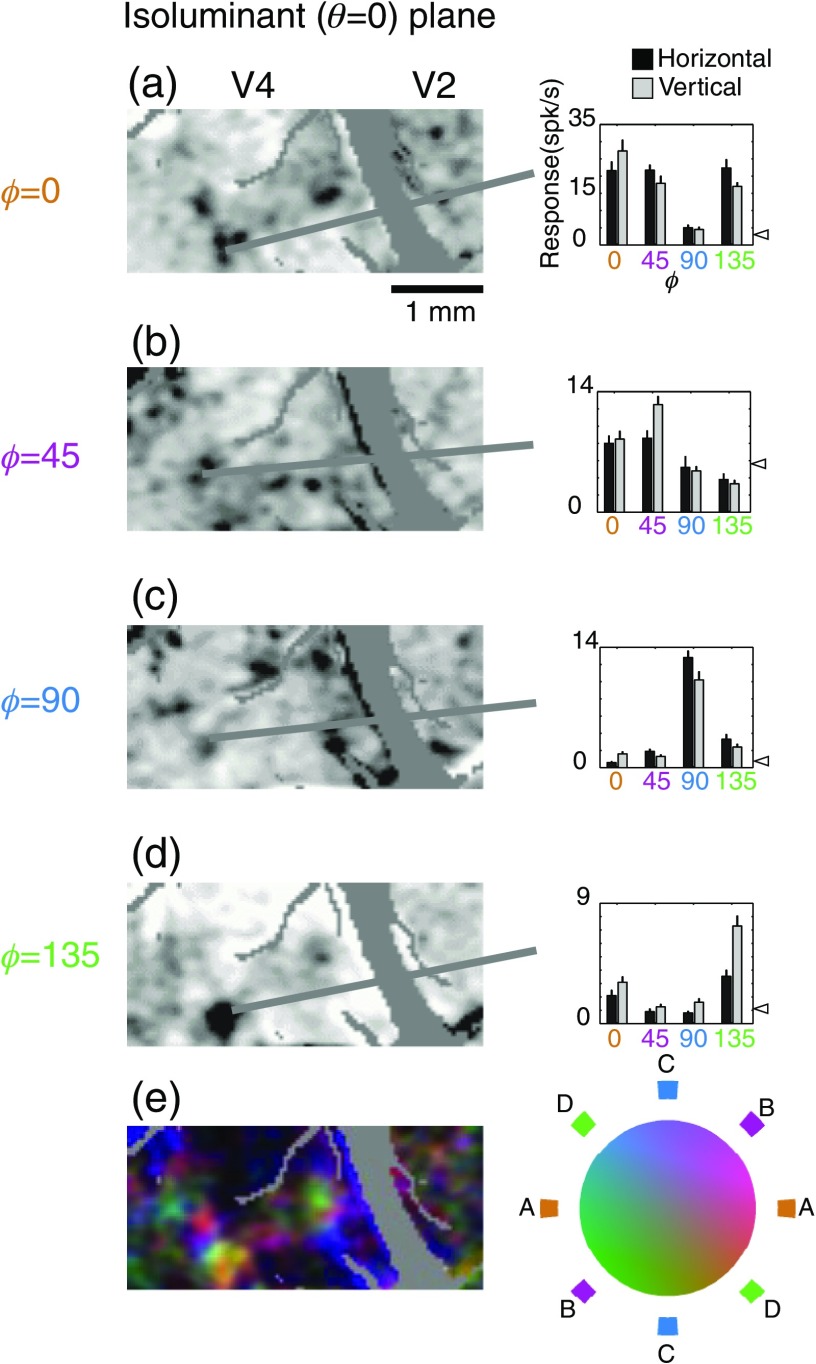
Isoluminant color-specific modules in V2 and V4 revealed by optical imaging (left) and verified by targeted electrophysiological recordings (right). Single condition maps for different axes of modulation in the isoluminant were constructed and verified with targeted single unit recordings (a–d) in which the lines indicate the location of the cell that was recorded on the map associated with its stimulus preference. The medium-gray outline in the figures describes a mask defined by the presence of large vessels visualized prior to the imaging session. The large gray diagonal corresponds with the lunate sulcus. Single unit responses with SE bars are shown to the right for both horizontal and vertical gratings modulated in the four axes in color space with spontaneous activity indicated by the triangles. While clusters for all four axes are clearly visible in V4, within V2 clusters along the cardinal axes (ϕ=0  deg and ϕ=90  deg) are the most visible.(e) shows a color-coded map incorporating all the single condition activation (a–d). Each single condition map was coded with a particular color as indicated by the band of colors surround the isoluminant disk in (e). For example, red-green activation (ϕ=0  deg,a) was coded in orange, and blue-yellow activation (ϕ=90  deg,c) in blue. The maps were then vectorially added to form a composite map of activation among isoluminant stimuli. In this composite map saturation indicates color selectivity; hue, color preference; and intensity, the strength of the signal.

**Fig. 3:**
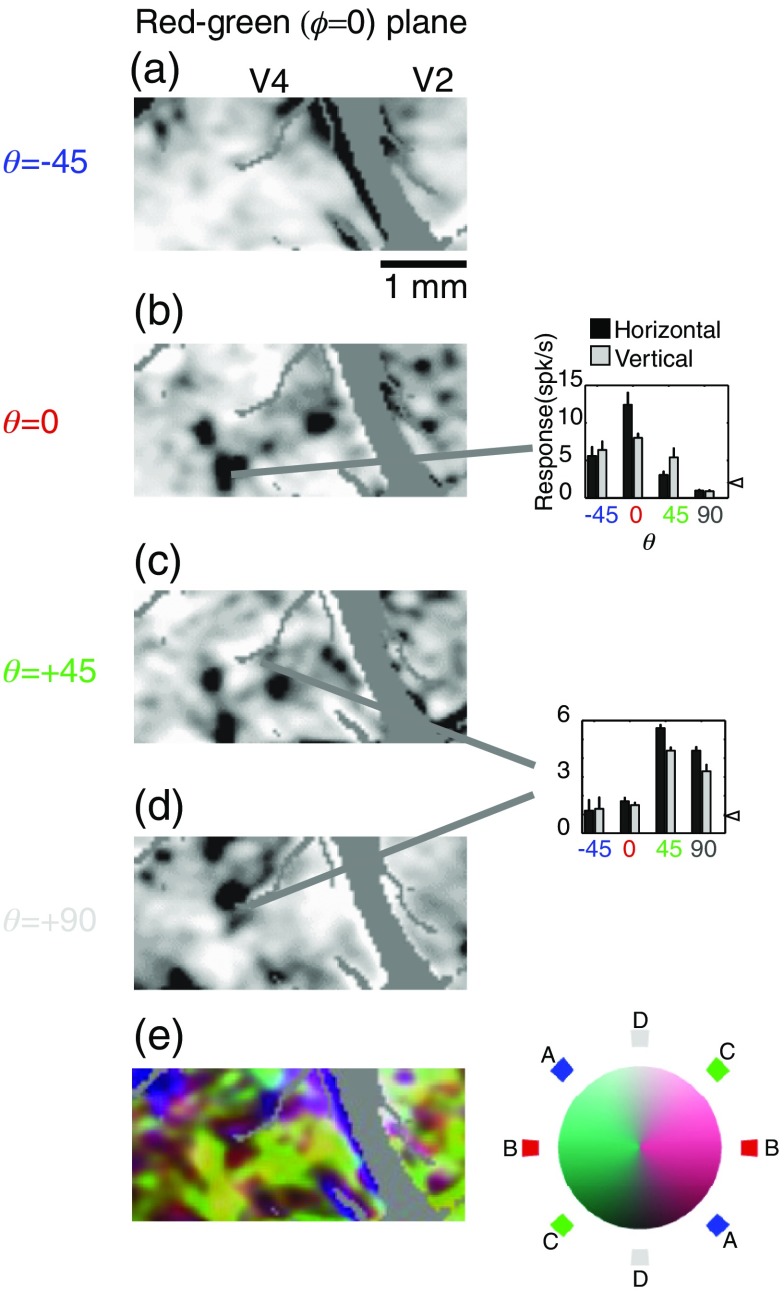
Red-green color-specific modules in V2 and V4 revealed by optical imaging (left) and verified by targeted electrophysiological recordings (right). Format is similar to that of Fig. 2, with single condition maps of stimuli modulated in different θ directions across the red-green (ϕ=0) plane (a:-45, b:0, c:+45, d:90) followed by a color-coded composite (e). Note that isoluminant responses in this plane are coded with red and achromatic responses in white. In this figure, blue and green represent responses to gratings that vary in both luminance and chromaticity (θ=−45  deg and +45  deg, respectively). The predominance of yellow in the composite map (e) indicates the colocalization of θ=45  deg and θ=0  deg responses.

**Fig. 4:**
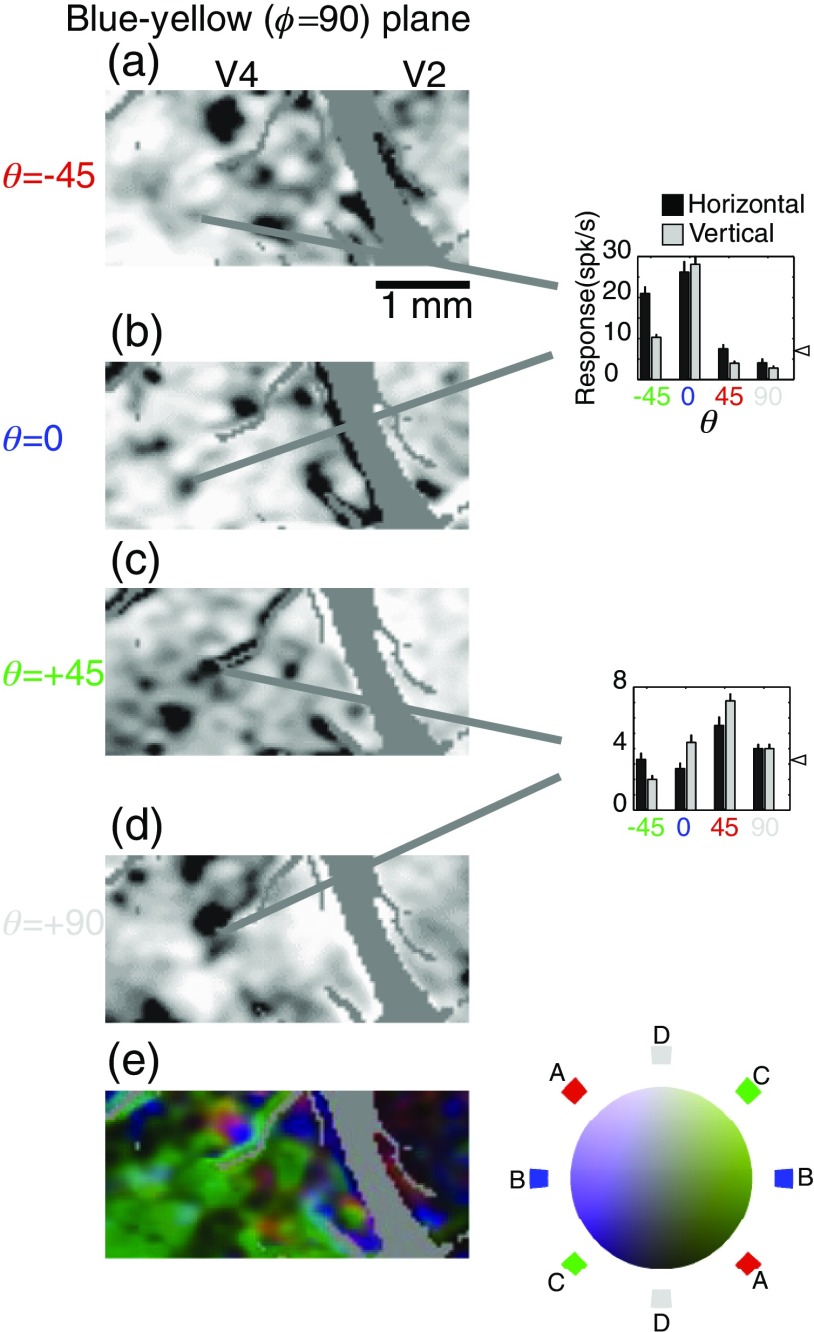
Blue-yellow color-specific modules in V2 and V4 revealed by optical imaging (left) and verified by targeted electrophysiological recordings (right). Format is similar to that of Fig. 2, with single condition maps of stimuli modulated in different θ directions across the blue-yellow (ϕ=90 ) plane (a:-45, b:0, c:+45, d:90) followed by a color-coded composite (e). Note that isoluminant responses in this plane are coded with blue and achromatic responses in white. In this figure, red and green represent responses to gratings that vary in both luminance and chromaticity (θ=−45  deg and +45  deg, respectively). Because the same achromatic luminance responses were used to form the composite of this figure and the previous figure, (d) of the two figures are identical.

Functional organization with respect to color was investigated by using stimuli that varied along nine different lines lying in three different planes within the color space [Fig. 1(a)]. In the three animals, we found clear evidence of color-specific patches of activation in both V2 and V4. Consistent with previous imaging^37^ and electrophysiological studies,^26^ color selectivity is not homogeneous across the areas, but is restricted to irregularly shaped domains. Figures 2–4 show the results from one recording session. In the isoluminant θ=0  deg plane (Fig. 2), different colors activate distinct punctate regions in both V2 and V4.

In V2, the strongest clustering is seen for colors along the cardinal axes (ϕ=0  deg and ϕ=90  deg). This parallels data from single cells in V1 in which most cells that preferred isoluminant stimuli had a preference along the cardinal axes.^40^ By contrast, in V4, clustering is readily apparent for all four axes in the isoluminant plane, consistent with a more uniform representation of color space.^44^^,^^45^ Finally, responses appear to be stronger (i.e., darker in the single condition maps and brighter in the composite map) in V4 than in V2.

If a given cortical area exhibited a strict visuotopic mapping and a small point spread function, a stimulus configuration such as the one we used would yield a patchy pattern of activation. This cannot explain the response maps we observed because such patchiness, in absence of explicit color organization, would be consistent between all axes in color space. Additionally, given the somewhat irregular topography of V2^46^ and V4^36^ and the considerable scatter in receptive field locations between nearby cells, we consider it unlikely that stimuli separated by a distance roughly corresponding with receptive field size would produce distinctly patchy patterns of activation in optical imaging. For example, in area V1, which has a much stricter retinotopic mapping than is seen in areas V2 and V4, point stimulation produces optically imaged activity with a space constant of several millimeters.^47^

Based on the magnification factor and receptive field size estimates by Gattass et al., the point image representation should span ∼4  mm in V4^43^ and about 2.5 mm in V2.^42^ As can be seen in Figs. 2–4, this space constant is much larger than the size of any feature we observe in the functional maps. Finally, because the cortical magnification in both V2 and V4 at this eccentricity is between 0.33  mm/deg and 1 mm deg,^42^^,^^43^ the entire imaged regions of V2 and V4 would only correspond to single stimulus patches [Fig. 1(b)]. These arguments indicate that any changes between the two areas in the pattern of activity elicited by our stimuli cannot solely result from differences in receptive field size or scatter.

To examine functional organization with respect to stimuli that are modulated in both chromaticity and luminance, we constructed maps in the ϕ=0  deg (Fig. 3) and ϕ=90  deg (Fig. 4) planes. In both of these planes, concentrated patches of activity within V2 are most visible in the isoluminant plane [θ=0  deg, Figs. 3(b) and 4(b)]. In contrast, patchy activation for stimuli off the isoluminant plane is clearly visible within V4 [Figs. 3, 4(a), and 4(c)]. There is clearly some degree of segregation with regard to changes in θ: panels A and C are neither identical, nor do they seem to reflect a simple summation of luminance (D) and isoluminant (B) activations.

**Fig. 5:**
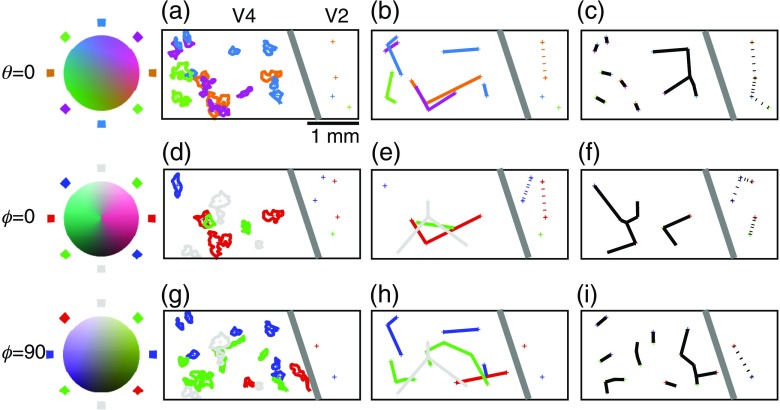
Geometric quantification of color-specific regions. Each row identifies regions activated by a particular direction along the color planes shown in the previous figures [(a-c): Fig. 2, θ=0  deg; (d-f): Fig. 3, ϕ=0  deg; (g-l): Fig. 4, ϕ=90  deg]. Contour maps on unfiltered single condition maps identify the borders of 70% maximal responses (a, d, g). Regions are colored according to the planes on the left in accordance with the codings used in the previous figures. Centroids are calculated for each region. Average radii are computed by averaging the distances from each vertex to the center point, which nearest neighbor distances by examining the distances between the centroids of different regions. Regions and distances within V4 indicated by solid lines; those in V2 by dashed lines. The second set of plots shows the lines connecting nearest regions of the same stimulus specificity (b, e, h). The third column of plots (c, f, l) shows the set of lines connecting the nearest regions of any stimulus specificity within the plane.

To quantify the number of color-selective regions and their layout, contour maps of the unfiltered reflectance data from each stimulus condition were constructed (Fig. 5). The color outlines of Figs. 5(a), 5(d), and 5(g) outline the darkest regions of Figs. 2–4: solid lines correspond to V4 regions and dashed lines correspond to V2 regions.

In V2, blue-yellow sensitive regions (ϕ=90  deg) are outnumbered by red-green sensitive regions (ϕ=0  deg) by a ratio of 3∶1. This is approximately the same ratio as was reported for color-selective regions within V1.^5^ Table 1 summarizes the numbers and geometries of color-specific regions observed in V2 and V4. When summing across all three animals the ratio was 11∶2.

**Table 1:** Geometry of color-specific regions in V2 and V4.

Stimulus		V2				V4			
θ	ϕ	N	Area ($\mu m^{2}$)	Radius (μm)	Radial symmetry	N	Area ($\mu m^{2}$)	Radius (μm)	Radial symmetry
0	0	11	30902±6608	106±12	0.15±0.02	10	42401±9214	120±13	0.15±0.02
0	45	0				23	27278±4932	108±11	0.17±0.01
0	90	2	10853±377	64±0	0.20±0.01	15	38970±14823	106±17	0.18±0.02
0	135	5	29793±8094	114±19	0.15±0.02	21	26390±3780	111±9	0.15±0.01
−45	0	9	25289±5726	100±10	0.15±0.02	19	25055±4175	98±7	0.19±0.01
−45	90	4	19927±2138	98±6	0.18±0.01	25	35883±6127	115±10	0.16±0.01
45	0	6	18899±2461	94±8	0.18±0.01	12	21033±2347	98±6	0.17±0.01
45	90	10	21665±2680	99±5	0.17±0.02	13	18677±2754	99±6	0.19±0.01
90		8	19498±2726	90±6	0.16±0.02	14	29375±8853	98±13	0.17±0.02

These data indicate that not just color preference but also color selectivity is localized to punctate regions. The geometries of these color-specific regions were quantified in two ways. Area, average radius, and circular symmetry were calculated for each region (Table 1). In this respect, no significant differences were observed between different stimuli or different visual areas (ANOVA): both V2 and V4 color-specific patches had a radius of ∼100  μm. Because our imaging window was not large enough to observe the periodicities on the order of millimeters that are suggested by anatomical^48^ and functional imaging^28^ studies of V2 and V4, describing the overall geometry and periodicity of color-specific regions was not feasible.

Instead we quantified the relative positions of response regions by looking at the vectors connecting nearest neighbors. Two sets of connecting vectors were computed: one connecting regions of identical stimulus selectivity [Figs. 5(b), 5(e), and 5(h)] and one connecting regions with different preferences along the same color space plane [Figs. 5(c), 5(f), and 5(i)]. Each connecting vector is described by a distance and a direction. The distribution of distances describes the spatial scale of clustering between different response regions, whereas the distribution of angles describes the consistency of the overall pattern of response regions. As was the case for the intraregion analysis, the interregion analysis revealed few differences between areas or stimuli (ANOVA). The variances of the angular distribution did not vary between visual areas (F-test), and both the same and different patch distances were similar. In all cases, the distances between patches of similar color preferences were on the order of 500  μm. The one significant difference (p<0.05) was the distance between isoluminant response regions (bold in Table 2), which was significantly smaller in V4 than in V2.

**Table 2:** Relationships between nearest neighbor color-specific regions in V2 and V4.

Stimulus		V2			V4		
θ	ϕ	N	Distance (μm)	Direction (deg)	N	Distance (μm)	Direction (deg)
0	Same	9	534±119	30±16	45	503±50	4±8
0	Different	13	412±62	29±15	43	249±25	12±8
Same	0	18	458±72	23±11	37	652±67	4±9
Different	0	20	305±53	−18±13	38	353±34	2±8
Same	90	11	456±98	25±11	44	469±54	−2±8
Different	90	15	423±121	26±12	47	294±24	1±8

Figures 3–5 show very little achromatic (θ=90  deg, panel D) selective activation in V2. Because these figures used cocktail subtraction, dark regions indicate selective activation. Therefore, the lack of achromatic regions indicates not the lack of response to achromatic stimuli, but rather the lack of a preferential response to achromatic stimuli versus all other color space stimuli. One concern, however, is that the relatively small area of V2 imaged in our V2/V4 exposures might have led to a preferential sampling of the color regions of V2. In this scenario, achromatic-selective regions were simply missed by our sampling window in all three animals. However, in separate experiments targeting the V1/V2 border (data not shown), in which a more extensive view of V2 was visible, very little achromatic-specific activation was seen in V2.

The lack of selective achromatic responses might be due to the specific characteristics of our stimuli. For example, regions of achromatic selectivity might appear with large field stimulation or with higher spatial frequencies. Our stimulus characteristics were chosen because, in our experience, they were well suited to revealing color-specific patterns of activation and activating single neurons in V4. The interactions between spatial frequency and color specificity are beyond the scope of this paper. However, we do know that our stimuli did not preclude the detection of orientation selectivity in single neurons of V4 [Figs. 2(d), 4(a), and 4(b)]. Moreover, these stimuli did not preclude the visualization of orientation stripes within V2.

It is still possible that these orientation stripes are not “normal,” i.e., that they do not correspond with the orientation stripes seen with the higher spatial frequency full field stimulation used in previous imaging studies. For example, if there were significant spatial frequency or surround interactions with orientation tuning in single cells, then the orientation map would be significantly altered. Alternatively, even if single neuron responses were separable, if there was strong functional organization for spatial frequency or surround effects then the orientation map would also appear different. However, our previous V2 optical imaging studies (data not shown) have shown that neither of these situations is applicable to V2 as the orientation maps are essentially unaltered over a spatial frequency range of at least several octaves (0.5 to 4  cyc/deg).

**Fig. 6:**
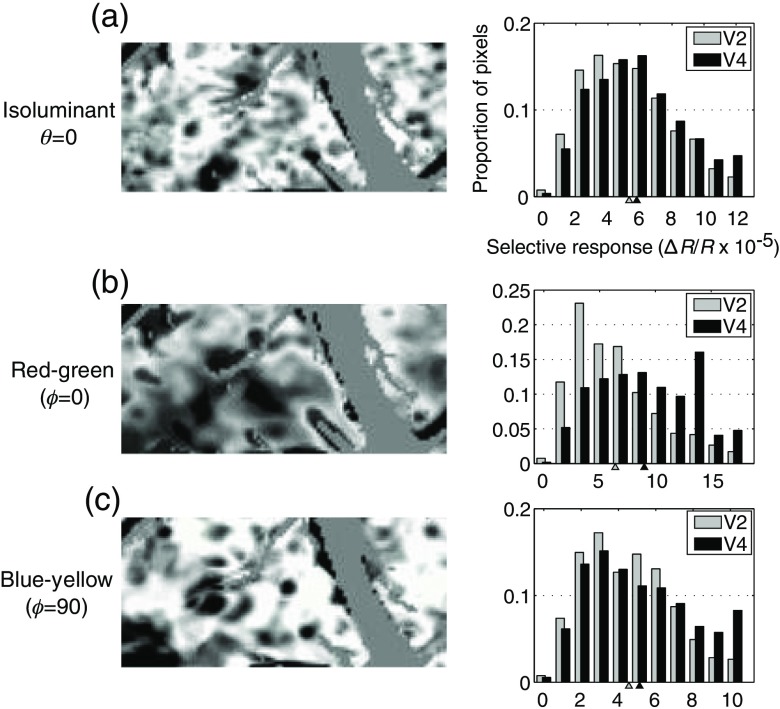
Selectivity maps across three different color-space planes [(a) θ=0, Fig. 2; (b) ϕ=0, Fig. 3; (c) ϕ=90, Fig. 4)]. In the images on the left, darkness indicates strong responses that are selective within the color plane. The corresponding panels on the right show the distribution of selective activation among V2 and V4 pixels. In all color planes, the selectivity is significantly higher in V4 (black) than in V2 (gray).

Figure 5 shows that, in both V2 and V4, there is relatively little overlap between regions of different color preference. To quantify the degree of color selectivity in the two areas, selective activation maps were constructed to show the spatial distribution of selective responses to stimuli within the three different color space planes [Figs. 6(a)–6(c)]. On the left panels, the spatial distribution of maximally selective and responsive regions is mapped, and in the right panels the distribution of selective activation between V2 and V4 is plotted. In all three color space planes, the average selectivity is significantly higher in V4 than in V2 (t-test, p<0.001). Although the magnitude of this difference is small, the pattern of consistently higher color selectivity in V4 compared to V2 was found in all three animals.

**Fig. 7:**
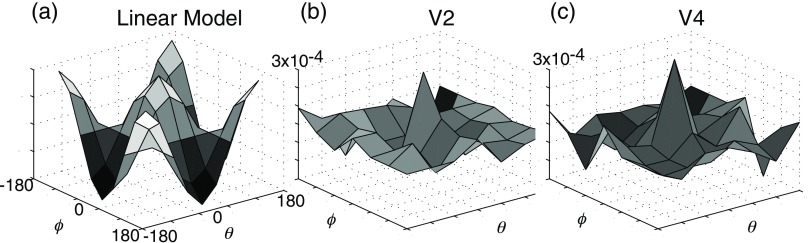
Average peak aligned color space tuning curves from the imaging data of Figs. 2–4. In (b) and (c), the average peak signal (ΔR/R) was $3\times 10^{-4}$. In neither V2 nor V4 is responses well characterized by a linear model that fits V1 electrophysiological observations. In both areas, the tuning is more selective than would be predicted by a linear model.

To examine the color tuning responsible for this selectivity, the color-space responses associated with each pixel in V2 and V4 were aligned with respect to their peak response and averaged. The resulting surface therefore indicates the degree of response selectivity in both θ and ϕ. In V1, single unit recordings have revealed that approximately half of the cells are well fit by a linear model of cone summation,^40^ consistent with a broad tuning in color space [Fig. 7(a)]. In contrast, the remaining cells exhibit nonlinear summation,^49^ which would suggest a sharper tuning in color space. Consistent with such cells, the average population tuning curves in both V2 and V4 are sharply peaked. Although our sampling was insufficient to measure precise bandwidths, the tuning curves of both areas are clearly nonlinear, with nonsinusoidal modulations in θ and ϕ. To establish this nonlinearity, a linear model was fit to the responses associated with each pixel. To quantify how well this linear model fit the observations, the mean square error of the model was divided by the variance of the responses in each pixel to yield a normalized error. No pixels in either V2 or V4 were well fit by linear models; the best fit pixels had a normalized error of 0.65. Although this reflects a nonlinearity in the relationship of single unit responses to the reflectance data, electrophysiological recordings also suggest a lack of linearity in color space: of the 23 V4 cells we tested, very few were well fit by a linear summation model. This nonlinearity is consistent with electrophysiological reports in both V4^50^ and V2.^23^^,^^51^ Moreover, they extend previous results by showing an increase in nonlinearity, as evidenced by a sharpening of color selectivity, between V2 and V4.

Figure 5 indicates the specific regions of color-specific activation, but it does not completely describe the distribution of activation seen in Figs. 2–4. For example, preference to a parameter might be localized to small regions, whereas weak nonzero activation for that particular parameter is very wide spread. In this case, no discrete regions would be discernible in the single condition map without cocktail subtraction. To address the overall distribution of activation while ignoring the particular spatial patterns shown in Fig. 5, we constructed population activation functions that look at the distribution of reflectance signal over an entire imaging region for a particular stimulus. Such distributions allow us to measure, for example, the proportion of an area that is half-maximally activated by a particular stimulus. This proportion is related to the typical bandwidth of selectivity for the various parameters describing the stimulus^52^ and the distribution of those stimulus selectivities across cells.

For example, if a few neurons responded to oriented drifting gratings such as we used in our experiments (i.e., they were narrowly tuned with respect to form), even if there was broad tuning with regard to color, only a limited proportion of neurons would be activated by any one of our stimuli. This possibility can be tested by looking at single-unit measurements of bandwidth. If the distribution of selectivity is not uniform such that only a small number of cells prefer a particular color, for example, the proportion of activated pixels will also be small. This notion might be examined by studying the stimulus dependency of activation width.

**Fig. 8:**
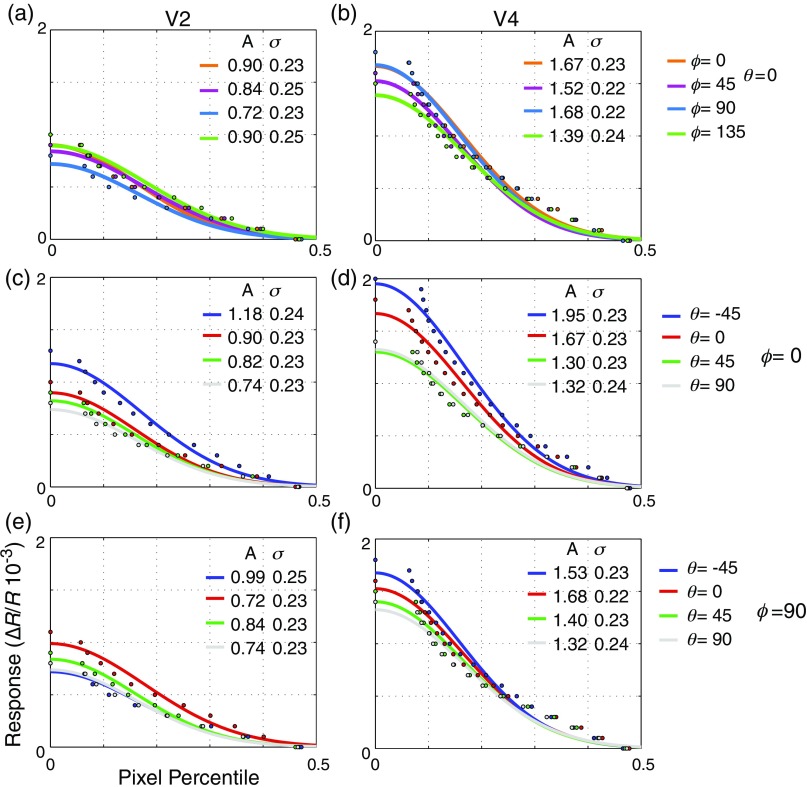
Color-specific population activity curves for V2 (left) and V4 (right) across three different color-space planes [(a), (b) θ=0, Fig. 2; (c), (d) ϕ=0, Fig. 3; (e), (f) ϕ=90, Fig. 4)]. The distributions of activity across cortical pixels for nine different axes in color space are plotted (filled circles) and fitted with Gaussians (solid lines) for V2 (703 pixels) and V4 (2201 pixels) separately. Each curve relates the percentage of pixels with responses greater than or equal to a criterion to the criterion chosen. Activity curves are grouped according to cortical area and color space plane. The parameters of the fitted Gaussians, amplitude A, and width σ are summarized in the insets of each panel. Despite the apparent stimulus dependency of functional organization evidenced in the previous figures, the curves show little variation in the overall distributions of activations. Consistent with the other animals in our study, these curves indicate a small decrease in the breadth of activation (σ) between area V2 and V4 that is associated with the increase in selectivity shown in Fig. 6.

Figure 8 shows the population activation functions for nine different axes in color space for the experiment shown in Figs. 2–4. The functions are based on the cumulative distribution of reflectances among pixels and, therefore, describe the magnitude and distribution of activity elicited by a particular stimulus. Functions were compiled for V2 and V4 separately for all three animals and all stimulus conditions. Each function was fitted to a Gaussian characterized by two parameters: amplitude and width (σ). The amplitude corresponds with the strongest activation seen; the width corresponds with the percentage of pixels with near maximal responses. The range of amplitudes (maximal activation) was from $5.7\times 10^{-4}$ to $2.0\times 10^{-3}$ and the range of σ was from 0.20 to 0.27. This range of widths corresponds to between 17% and 22% of pixels having at least half-maximal activation. Most of the variability in these parameters is due to differences between animals and visual areas and is not stimulus specific; as can be seen in Fig. 8, the range with a single color space plane and visual area is considerably smaller.

To examine how the distribution of stimulus activation parameters varied according to stimulus and between areas, we performed a two-factor ANOVAs. This analysis revealed significant differences in both amplitude (p<0.05) and width (p<0.001) between area V2 and V4, but no significant effect of stimulus and no interaction between stimulus and area. In all three animals, activation widths were consistently smaller in V4 than in V2: repeated measure ANOVA revealed no significant interanimal variability in the interareal change of activation width. Although consistent, the actual change in width was usually small: the greatest interareal σ change was from 0.26 to 0.21, and the average change was 0.02. This is consistent with the small but significant increase in color selectivity shown in Fig. 6. However, for amplitude, there was interanimal variability (p<0.001). In the two animals with parafoveal cortical exposures (used to construct the population activation curves of Fig. 8), maximal activation was significantly larger in V4 than in V2. In the third animal, which had a more foveal exposure [Figs. 12(c) and 12(d) versus 12(a) and 12(b)], maximal activation was stronger in V2. Thus maximal activation strength is likely to reflect parameters that were varied between different eccentricities, specifically the size and spacing of the grating patches used for stimulation.

The apparent lack of stimulus dependency in these population measures is notable given the visible differences between different color stimuli shown in Figs. 2–4. For example, in the isoluminant plane (θ=0  deg) patches in V2 are more visible for the stimuli along cardinal axes (ϕ=0  deg and ϕ=90  deg) than for off-axis stimuli [Figs. 2(a) and 2(c) versus 2(b) and 2(d), 5A, Table 1], yet the population activation curves look remarkably similar [Fig. 8(a)]. Similarly, V2 achromatic activation (θ=90  deg) is similar to the activation seen with other directions in color-space, despite the relative absence of achromatic-specific regions (Figs. 3–5). It should be emphasized, however, that these population activity measures ignore both selectivity and spatial organization: unlike the functional maps, the activation functions cannot distinguish distinct populations of color-specific response from populations of responsive, but unselective, neurons. These measures demonstrate that even in cases where the distribution of responses over very large regions (on the order of ${\mathrm{mm}}^{2}$) is similar between areas, the areas can be readily distinguished on the basis of the response organization on the scale of microns. Close inspection, however, suggests that these measures are not completely insensitive to changes such as those seen in Fig. 2. Looking at the 18 width measurements in Fig. 8, for all cases in which σ is 0.24 or larger (V2 θ=0  deg, φ=45  deg; θ=0  deg, φ=135  deg; θ=−45  deg, φ=0  deg; θ=−45  deg, φ=0  deg) patches are not readily visible in the functional maps [Figs. 2(c), 2(e), 3(b), and 4(b)]. Thus from the population activity data, we can conclude that clear changes in functional organization can be associated with subtle changes in total population response: even in the absence of clear functional organization for a particular parameter, a considerable proportion of neurons may be responsive to that parameter.

**Fig. 9:**
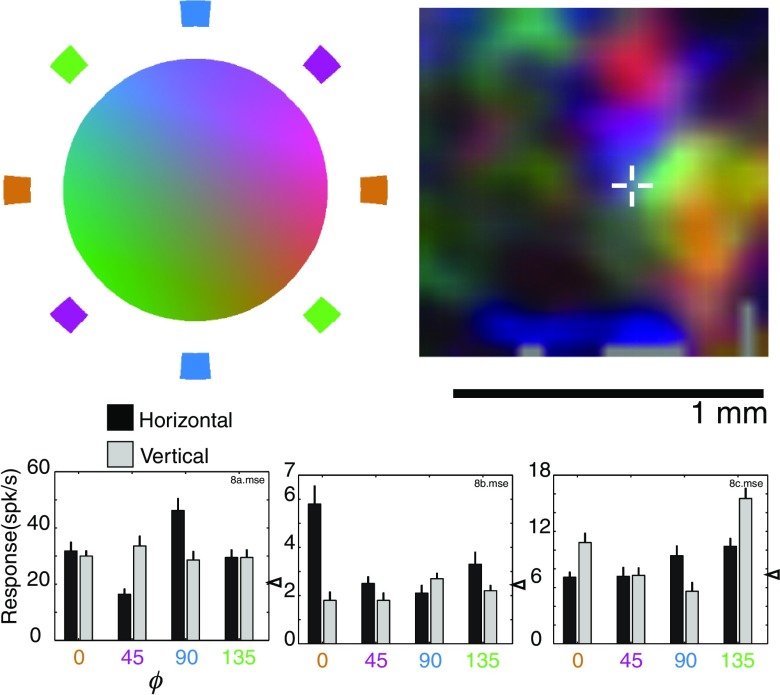
Columnar properties near the edges of isoluminant color modules. Isoluminant color map is an expanded view of a region shown in Fig. 2. Single unit recordings at 100  μm intervals along a vertical penetration reveal a diversity of color-selective responses not observed in penetrations that are well centered on color-selective patches. Spontaneous activity is indicated by the triangles.

Single unit recordings support this conclusion. Figure 9 shows one example of three supragranular cells from a vertical penetration near to several color-selective patches, but not centered on any one of them. Although the cells display color-selective responses, they have different preferences. We only observed such variations when penetrations were not well-centered on color-selective patches. These variations further emphasize the difficulty in studying functional organization with classical electrophysiological techniques: even a slight change in position would result in very different observations about the physiological consistency of nearby cells.^26^

### 3.2 Color and Size Maps

In a previous study, we reported functional organization with respect to stimulus size in both areas V2 and V4.^36^ We termed regions that preferentially responded to small stimuli “S regions,” and found that while in V2 they were largely segregated from regions of maximal orientation selectivity, no such segregation was apparent in V4. In order to study the relationship between color organization and size organization in the two areas, we ran successive imaging sessions in which first color and then size was varied. For the color analysis, the standard stimuli were used (Fig. 1).

For the size analysis, images obtained during stimulation by an array of small patches of about 1.5 deg in size were compared to images in which full screen stimulation was presented. In the size run, two types of gratings were used: an achromatic square wave and a square wave that included both red-green and luminance modulation. Separate maps for size preference were constructed by comparing images obtained with full-screen stimulation with images obtained during the two kinds of spot stimulation. S regions, in which small stimuli were preferred, were plotted by constructing isoresponse contours for each differential map.

**Fig. 10:**
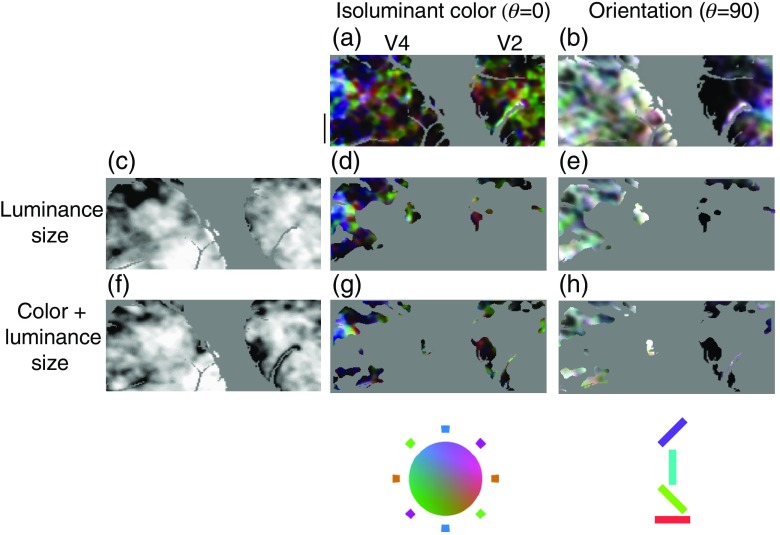
Overlap among color, orientation, and size selectivity in V2 and V4. The isoluminant color map (a) was constructed using stimuli as detailed in Fig. 1. The orientation map was constructed using achromatic oriented gratings (b). Size maps were constructed by comparing stimulation evoked with an array of small stimuli with full-field (22 deg) stimulation. Two types of stimuli were used for the size maps: one which contained both chromatic and luminance modulation (f), and one which only contained luminance modulation (c). Contour lines were plotted to indicate the outline of S regions in which preferential responses to small stimuli were observed. These S regions were used to mask the color and orientation map, so that (d) and (g) indicate color selectivity within regions that preferred small stimuli while (e) and (h) only orientation selectivity within such regions. In V2, there is little orientation selectivity within S regions, as indicated by blackness of S regions in V2. On the other hand in V4, there is considerable orientation specificity within S regions, as indicated by the coloration of V4 S regions.

In order to reveal overlap between size selectivity and color and orientation selectivity, the S regions were used to mask color [Fig. 10(a)] and orientation [Fig. 10(b)] maps. Figure 10 shows overlap between size and color [Figs. 10(d) and 10(g)] and size and orientation selectivity [Figs. 10(e) and 10(h)]. The absence of selectivity in orientation-size maps in V2 indicates a complete segregation of these selectivities consistent with our previous report. This is in contrast to S regions within V4 in which orientation selectivity is visible. Thus while size selectivity is largely segregated from orientation selectivity in V2, this functional segregation disappears at the level of V4.

**Fig. 11:**
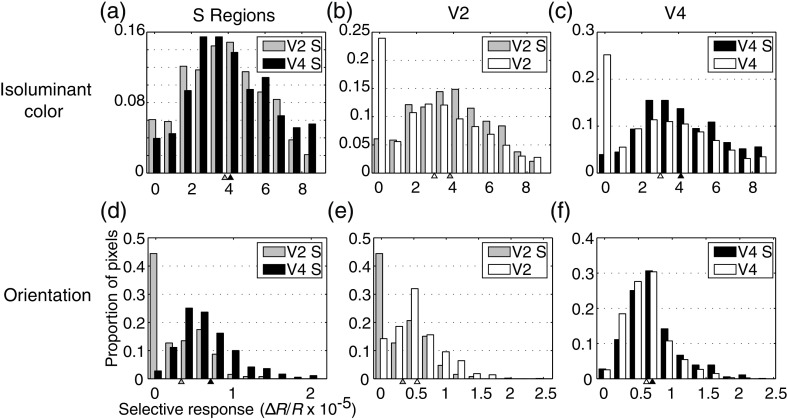
Distribution of color and orientation selectivity within S regions of V2 and V4. Color (a) and orientation (d) selectivity is significantly higher for S regions within V4 (black) compared with those in V2 (gray), although the difference in color selectivity (a) is small. Color selectivity is positively correlated with size tuning in both (b) V2 and (c) V4: in both areas the selectivity is larger within S regions [gray versus white in (b), black versus white in (c)]. The difference between S region orientation selectivity in the two areas is considerably larger than that seen with respect to color selectivity: over 40% of S region pixels in V2 were unselective for orientation, while less than 5% were in V4 (d). This is due to a change in correlation between size and orientation selectivity: in V2 S region orientation selectivity is significantly lower than orientation selectivity overall (gray versus white in e), while in V4 there is no such difference (black versus white in f).

In order to quantify the degree of segregation shown in Fig. 10, we computed the selective activation by isoluminant color and orientation within the luminance S regions of V2 and V4 (Fig. 11). Although only the S regions defined by luminance stimulation are shown in this figure, the results were identical for S regions defined by the color+luminance stimulus [Figs. 10(f)–10(h)]. The left panels show the distribution of color (A) and orientation (D) selectivity within the S regions of V2 and V4. In both cases, V4 S regions show significantly higher selectivity (t-test, p<0.001). Although the difference between the areas is small with respect to color selectivity, there is a large difference with respect to orientation selectivity: over 40% of S regions pixels are not orientation selective in V2, while less than 5% of V4 S regions pixels are unselective for orientation [Fig. 11(d)]. Any differences between S regions of the two areas might have two causes: a difference between the areas overall or a difference in the spatial segregation of selectivity between the areas.

Figures 11(b) and 11(c) show that for color selectivity the difference between V2 and V4 S regions is largely due to differences between the areas overall: as noted previously, V4 has slightly higher color selectivity than V2 in all three animals. In both V2 and V4, color selectivity is significantly higher (t-test, p<0.001) within S regions, indicating a positive correlation between size and color selectivity in both areas and therefore little difference in the segregation of size and color information between the areas. On the other hand, the difference seen between S region orientation selectivity in V2 and V4 [Fig. 11(d)] suggests a reduction in the degree of segregation between size and orientation in V4. In V2, S regions show significantly less orientation selectivity than V2 overall (t-test, p<0.001): while over 40% of S region pixels were unselective for orientation, only about 10% of pixels throughout V2 were completely unselective for orientation [Fig. 11(e)]. By contrast, there is no significant difference between S region and overall orientation selectivity within V4 [Fig. 11(f)]. This difference with respect to orientation confirms the findings of our previous report:^36^ virtually all V2 S regions pixels were unselective for orientation, while in V4 there is an independence between size and orientation selectivity such that the majority of S region pixels were selective for orientation.

### 3.3 Color and Orientation Maps

**Fig. 12:**
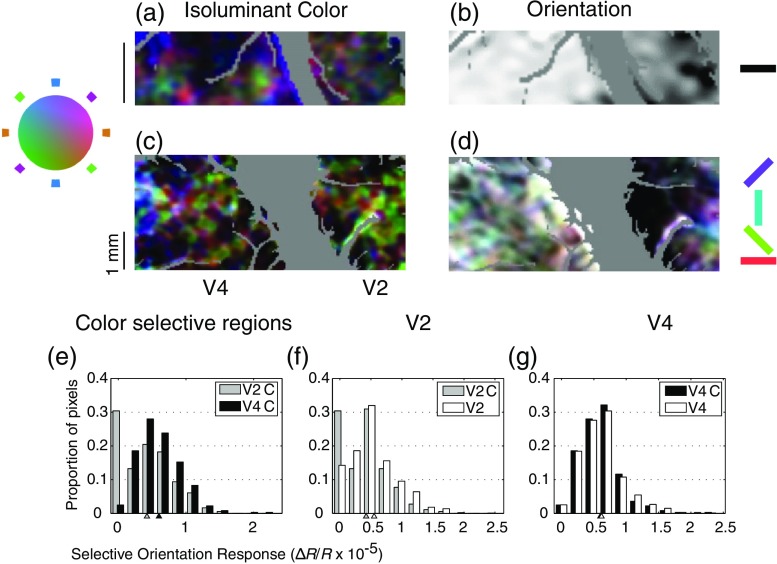
Organization for orientation and color as a function of eccentricity. Isoluminant color maps were constructed by summing across images obtained with both horizontal and vertical stimuli. Orientation maps were according to achromatic stimulation. Two animals are shown. In (a, b), the cortical exposure corresponded with parafoveal representations in V2 and V4. For such exposures, organization with respect to color is visible in both (a) V2 and V4, but organization with respect to orientation is solely visible in area (b) V2. In (c, d), a more dorsal and lateral cortical exposure revealed more central representations in V2 and V4 in which organization with respect to orientation was visible for (d) both areas. In V2, there is only partial overlap between orientation selectivity and color selectivity, as indicated by the lack of orientation selectivity seen in many color-selective regions. (e) In V4, however, there is extensive overlap between the orientation and color maps. This is quantified by computing the distributions of orientation selectivity among color selective pixels separately for V2 (gray) and V4 (black). In V2, more than 30% of color-selective pixels did not exhibit orientation selectivity indicating a segregation between color and orientation. In contrast, in V4 less than 5% of color-selective pixels were not orientation selective. Just as with size (Fig. 11), this areal difference reflects a change in segregation of selectivities: in V2 orientation selectivity is significantly lower in color-selective regions (gray versus white in f), while in V4 there is loss of orientation selectivity among color-selective regions (black versus white in g).

The presence of organization for both orientation and color in V4 allows us to examine the relationship between the functional maps for these attributes in V4. Figures 12(c) and 12(d) show functional organization for isoluminant color and orientation, respectively, for the same region shown in Fig. 10.

As with previous figures, the color space map was constructed by summing the activation seen for both horizontal and vertical gratings. The orientation map was constructed using achromatic stimulation (θ=90  deg). In V2, orientation-specific domains align in stripe-like formations whereas in V4 no such geometry is visible. Although there is some degree of overlap between color-specific and orientation-specific regions in area V2, the overlap is considerably more apparent in V4. The segregation between color and orientation is quantified in Figs. 12(e)–12(g), by looking at the distribution of orientation selectivity for color-selective regions in V2 and V4. Color-selective regions were defined according to selectivity maps as shown in Fig. 6: pixels whose selectivity was within 70% of maximal selectivity were defined as selective.

In V2, over 30% of such color-selective pixels were not orientation selective, while in V4 less than 5% of such color-selective pixels were not orientation selective [Fig. 12(e)]. Just as with the overlap between size and orientation, this areal difference is significant (t-test, p<0.001) and is due to a desegregation of orientation and color in V4: while orientation selectivity is significantly lower within color regions of V2 [Fig. 12(f)], this is not the case for V4 [Fig. 12(f)]. These results are consistent with electrophysiological surveys of the two areas indicating a partial segregation of col6or and orientation specificity in V2^22^^,^^23^ and no segregation in V4.^30^

**Fig. 13:**
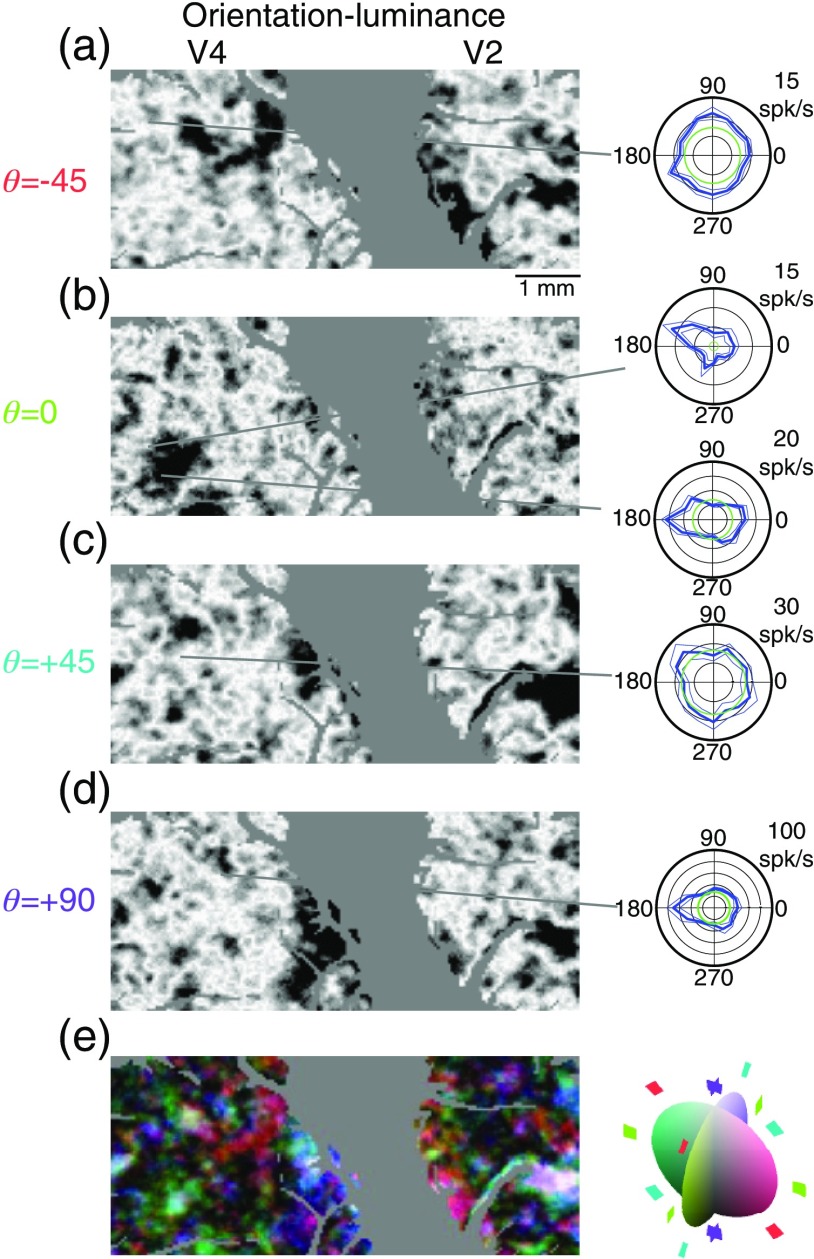
Interaction between color and orientation selectivity. (a–d) Single condition maps were composited to form (e) a map describing how orientation selectivity varies with luminance modulation. Polar plots indicate response rates of single cells at locations indicated by gray lines to grating patches of various orientations. In these plots, the thick line is the mean response, thin lines means SE, and the green line means the spontaneous activity. (a–d) Single condition maps were formed by taking the absolute value of the difference between images obtained with horizontal stimulation and images obtained during vertical stimulation. Dark pixels therefore represent regions in which the responses to horizontal and vertical stimuli were maximally different. In (e), saturation indicates selectivity in color space; regions that are equally selective for all axes in θ appear white. (b) Unlike V4, V2 has very limited regions of orientation selectivity when isoluminant stimuli are used. Regions of orientation selectivity change far more drastically as a function of luminance modulation in area V4 than in area V2 (e.g., a versus c); (e) there is only one small colorless region in the color map of V4.

To directly examine the consequences of changes in overlap between the areas, we constructed functional maps of orientation selectivity as a function of θ in color space (Fig. 13). In this case, the single condition maps of orientation selectivity were computed by taking the absolute value of the difference between images obtained with horizontal stimuli and images obtained with vertical stimuli. As shown in Fig. 12, V2 in contrast to V4 has very limited regions in which orientation selectivity is visible for isoluminant stimuli. In V2, the primary region of horizontal-vertical selectivity for achromatic stimuli [Fig. 13(e)] is also orientation selective for θ=45  deg and θ=−45  deg stimuli, while in V4 the location of orientation-selective patches clearly depends on θ. These results demonstrate the emergence of functional organization with respect to a higher-order selectivity (orientation-color) as a result of the overlap between functional domains defined by more primitive selectivities. The observation that in V4 orientation selectivity can depend on color is corroborated by our single-unit observations. For example, in the cell shown in Fig. 3(b) (right), the preference between horizontal and vertical reverses between θ=0  deg and θ=45  deg while the cell in Figs. 4(a) and 4(b) (right) only exhibits a horizontal orientation preference for θ=−45  deg. Figure 13 shows that such interactions are not idiosyncratic, but rather that they are characteristic of specific regions within V4.

## 4 Discussion

By using the optical imaging technique, we have directly examined and compared the functional organization to two cortical areas of the ventral pathway, V2 and V4. While this technique alleviates some of the sampling concerns of single unit electrophysiology, it is important to recognize that there are still limits to this methodology. For example, we have not exhaustively sampled the potential stimulus space to which neurons in the two areas might respond. Specifically, we have sampled responses to a limited number of points in color space using horizontally and vertically oriented stimuli of fixed position, size, and spatial frequency. Although we did not systematically explore these other parameters, significant deviations in these parameters often resulted in minimal activation in imaging maps consistent with single unit physiology. For example, in accordance with previous electrophysiological studies, large full-screen (usually around 20 deg) stimulation is not optimal for most V4 neurons. Similarly, in our experience and consistent with previous reports, neurons with higher optimal spatial frequencies tend not to exhibit color selectivity. The visibility of large amount of activation throughout our optical maps suggests that, although we necessarily did not sample responses to all possible visual stimuli, our stimulus set was comprehensive enough to elicit activity in the majority of cells. Moreover, the presence of normal orientation maps in V2 with our low spatial frequency arrayed stimulation suggests that our choice of stimulus does not preclude the visualization of classically observed patterns of functional organization such as the stripes of V2.

Although our imaging method samples large numbers of neurons over square millimeters of cortical surface, we are necessarily limited to those portions of the areas that are externally visible. If functional organization varies significantly within an area, then our results might not be applicable throughout the entirety of areas V2 and V4. However, there are few reports if any of anatomical or physiological properties being unusual in the regions of V2 and V4 surrounding the lunate. In V2, cytochrome oxidase (CO) bands usually extend all the way to the V1-V2 border,^53^ and the full complement of functional stripes has been observed in postlunate V2 through both electrophysiology and optical imaging.^46^ In V4, almost all electrophysiology has included the prelunate surface, and neither intrinsic horizontal connections^54^^–^^56^ nor extrinsic connections^48^^,^^53^ appear unusual.

Because the regions we studied are activated simultaneously by the same stimuli, our experiments reveal how two cortical areas represent equivalent stimulation of a region of visual space. With this technique, we previously demonstrated organization with respect to orientation and size in the two areas. Here, for this first time, we have extended those results by systematically exploring a physiologically defined color space and finding functional organization with respect to color in both V2 and V4. Several differences exist between V2 and V4: V4 contains a more elaborate representation of isoluminant color space than does V2, and color activation is limited to a smaller population than V2. Unlike V2, functional organization for the attributes of color, size, orientation is not highly segregated in V4. Rather, functional domains for different visual parameters extensively overlap in V4. In conjunction with comparisons between V1 and V2, these results suggest that higher visual areas are characterized by a progressive desegregation or integration of functional domains for simple stimulus attributes. This integration might be necessary to construct higher-order visual representations supporting object recognition.

### 4.1 Anatomical Correlates of Functional Organization

The concentration of long-range horizontal connections in the supragranular layers into patchy formations appears to be a universal feature of cortex: they have been found in visual areas V1, V2, V4, 7a,^57^ somatosensory areas 3b, 1, and 2, and motor area 4.^56^ In area V4, the round or oval patches revealed by both retrograde and anterograde tracer injections have a width from 250 to 500  μm with a spacing of around 450 to 1300  μm.^54^^–^^56^ Although our measurements of functional domain size tend to be slightly lower than these anatomical estimates, this might be due to the particular response criterion used in defining the “borders” of regions. Since the distribution of activity to any given stimulus is a continuous function (Fig. 8), the choice of the criterion is somewhat arbitrary. However, because our criterion was consistent for all experiments, we are able to compare domain size between the areas and different stimuli. Our result that color-specific regions are largely circular and similar in size between the areas is consistent with the small changes seen in anatomical patch size and geometry between the two areas. However, we found no increase in functional patch spacing to correspond with the increase in anatomical interpatch distance seen in V4.^54^^–^^56^ Although this discrepancy suggests a lack of correspondence between intracortical connectivity and color selectivity, it may simply be the result of our limited spatial window precluding the observation of large interpatch distances in V4.

Anterograde injections in V2 suggest large projection regions in V4 of around 5 mm.^48^ Although our imaging window (typically around $8\;{\mathrm{mm}}^{2}$) limits our ability to estimate the total spatial extent of functional domains, the presence of features that are several mm in extent (e.g., the isoluminant responses seen in Fig. 2) is suggestive that patterns in functional organization may have anatomical correlates in such divergent interareal projection patterns.

Although Hubel and Livingstone^22^ proposed a tight association between receptive field properties in areas V1 and V2 and metabolic activity, as assessed by CO staining, more recent studies are more equivocal.^46^^,^^48^^,^^58^^,^^59^ For example, pale and thick stripes in V2 as defined by CO, and associated with orientation and stereo/motion selectivity, draw from the same interpatch (CO-low) inputs in V1^60^ and regions in V4 draw from both thin and pale stripes in V2.^48^^,^^61^ Finally, no obvious correlations between CO staining and functional properties have been reported in V4.^62^

### 4.2 Color Processing

Recent reports of color properties in visual cortex on the basis of single cell recordings reveal little consistency: the estimates of the incidence of color cells in V4, for example, vary widely.^14^^,^^15^^,^^25^^,^^63^ One reason for the disparity in results is the variety of stimuli applied, and criteria used, to evaluate color selectivities. For example, color preference and opponency have often been evaluated qualitatively or with stimuli of different dimensions or spatial frequencies. As would be expected, the variety of results even within visual areas has led to a variety of conclusions regarding the progression of color processing in different areas. For example, V4 has been proposed to be the final stage in color perception, and essentially undistinguished from earlier visual areas with regard to color selectivities.^17^ As mentioned in Sec. 1, limited sample sizes are also problematic, especially when, as we have demonstrated, the distribution of color properties is not uniform.^41^ This issue becomes worse with the possibility that the specific patterns of functional organization might vary between individual animals. Thus, the sampling issues inherent in the classic method of interareal comparison are further exacerbated by intraareal differences and interanimal differences.

Our optical imaging approach alleviates these concerns by examining the color properties of large numbers of neurons in the multiple areas with consistent stimulation.^64^ Our stimuli are described by a color space that has been used to quantitatively characterize color responses at a variety of stages from the LGN to V2. This choice allowed us to making direct comparisons between V2 and V4 and to draw inferences regarding the progression of color properties from the geniculate to extrastriate cortex. This is especially important because certain studies of color properties in V4^16^^,^^30^ have relied upon monochromatic stimuli that are not readily comparable to the V1 and V2 data, or hue variations that may not sample color space systematically.^37^

Our data reveal that V4 responses are slightly more selective in color space than those of V2. Population activity functions that pool stimulus responses across locations also reveal an areal difference: stimulus responses to our color stimuli were slightly more narrowly distributed in the V4 population than in the V2 population. This change in activation could be due to two factors: a decrease in the number of neurons that respond to drifting gratings or a narrowing of the bandwidth of selectivity. For V4, we can assume that about 75% of neurons respond to simple gratings.^65^ Unfortunately, color bandwidth data for V4 are largely unavailable. If we assume that this bandwidth is about one quarter of the range,^41^ we get a σ of 0.23 which is consistent with our observations. The difference between V2 and V4 was only 0.02 or a decrease in the size of half-maximal activity population of 2.3%. Although our sampling in color space was insufficient to quantitatively evaluate bandwidths of tuning, the increase in color space selectivity seen within V4 suggests that V4 cells tend to have smaller bandwidths than V2 cells (Fig. 6).

In the LGN, there is a bimodal distribution of color selectivities among cells in the parvocellular layers along the cardinal color planes of the DKL color space.^38^ In V1^40^ and V2,^23^ there is no such distribution: the distribution of optimal azimuths (φ) in the color space is not strongly concentrated along these cardinal planes. However, if the analysis is restricted to cells that preferentially respond to isoluminant stimuli (θ near 0), the bimodality is still present among V1 cells. The population activity functions show that the overall breadth of activation in both V2 and V4 is similar for different axes in color space. The difference in color space selectivity, although significant, is small. This would suggest that the areas cannot be readily distinguished according to color response properties. Given the relative coarseness of our color space measurements, it remains possible that some aspects of color responses, such as the exact nature of the nonlinearity in color tuning, are notable different for the two areas. In particular, although V1, V2, and V4 all exhibit a reduction in overt chromatic opponency, electrophysiological evidence suggests a progressive decrease in opponency such that color-space direction selectivity more developed in V4 in comparison to earlier areas.^66^ Because all the stimuli used in this study were symmetrically modulated around the equal energy point, the degree of functional organization with respect to different directions along the same axes in color space remains an open question.

Although a more thorough examination of color space might reveal more differences between V2 and V4, it seems unlikely that it could eliminate the differences that are visible with our measurements. Specifically, our functional maps demonstrate that the areas can be distinguished on the basis of how selective color responses are spatially organized. As shown in Fig. 2 and Table 1, localized regions of isoluminant-specific response in V2 are largely limited to red-green stimulation. The discrepancy between the red-green preference indicated in the selectivity analysis and the lack of any obvious preference in the activation analysis suggests that the bandwidth of color selectivity in V2 strongly depends on hue. In any case, this red-green preference is not true in V4, where robust isoluminant color-specific regions are visible at all orientations in the isoluminant plane [Figs. 2(c) and 2(e), Table 1]. Since human psychophysics suggests the presence of channels tuned to different isoluminant chromatic axes,^67^ the diversity of isoluminant selectivity in V4 might play an important role in the elaboration of color representation.

Although functional domains, such as regions responsive to isoluminant stimuli, are irregularly spaced, they do not appear to be randomly distributed in V4. For example, examining Figs. 2(a), 12(c), and Table 2, we see that isoluminant-specific response regions are more closely aggregated in V4. This consistent with fMRI studies that have revealed large mm-sized regions of color selectivity within V4^28^^,^^64^ which have termed “globs.” Whole brain functional imaging reveals that such globs are present in a variety of visual areas including V4 and posterior inferior temporal cortex^68^ and suggests that a progression of color selectivity from a cone space in the LGN^38^ to perceptual hue space as one ascends the visual hierarchy.^44^^,^^69^ Our results are consistent with such a proposal: we find a greater diversity of DKL preferences and an increase in color nonlinearities as one goes from V2 to V4. However, our finding of V4 selective regions in the theta plane in which luminance modulations are present is consistent with electrophysiological recordings suggesting that the transition to a hue (and luminance-independent) representation is far from complete at the stage of V4.^70^

We show that V4 “globs” have a color-specific substructure. The proximity of different color-selective regions allows for the study of an interesting aspect of functional organization that, before this report, has only been shown in primary visual cortex: the presence of pinwheels and fractures at which sudden changes in functional preference occur. Although our study of such regions was limited, Fig. 9 suggests that such regions are characterized not by a lack of selectivity in single units, but rather a mixture of cells with different color selectivities. This is analogous to the finding of normal orientation tuning in the pinwheel centers of cat V1.^71^ In V1, discontinuities in orientation selectivity are correlated with discontinuities in the visuotopic representation.^72^ Given the lack of precise retinotopy in V4,^43^ it seems unlikely that such a correlation could be observed in V4. However, it is possible that discontinuities in different functional domains, such as color and orientation, are related. Anecdotal support for this hypothesis can seen in the single unit physiology of Fig. 9, in which a very crude measure of orientation selectivity, the ratio of horizontal to vertical responses, seems to vary both between cells and color.

Such correlations between different functional domains could only be present if there were substantial overlap between different domains. Our data suggest that such overlap is more prevalent in V4 than in V2. As shown in Fig. 12, our data suggest considerable segregation between color and orientation selectivity in V2. Hubel and Livingstone^22^ suggested a fairly strict segregation of the two selectivities, reported as a paucity of oriented cells with color coding. Their recordings in both squirrel monkey and macaques did not explicitly test color properties among oriented cells and used a different scheme for the classification of color selectivity.

Studies using the same color space employed in our study have found that about half of color-selective cells were not orientation selective, whereas the total incidence of cells lacking such selectivity was 25%.^23^^,^^24^ Our V2 data show the same trend: 30% of color-selective regions have no orientation-selective activation compared to about 15% overall [Fig. 12(f)]. The difference between our incidences and those published previously might be due to either different criteria or our use of lower spatial frequencies. In any case, Fig. 12(g) shows that the overlap in V4 for the same stimulus is significantly more substantial, consistent with electrophysiological reports of a lack of relationship between the incidence of color and orientation selectivity.^30^

The data from this paper and our previous study^36^ also suggest a segregation between orientation and size selectivity in V2. Although an electrophysiology survey^24^ failed to find any significant anticorrelation between orientation and end-stopping, this difference may be a result of its limited sample size (end-stopping: 22 cells, orientation selective: 82) and differences between end-stopping and size tuning.^33^ The overlaps visible to a limited extent in V2, and to a considerable extent in V4, argue against a strict segregation of color and form processing and are consistent with psychophysical results showing equivalent performance between tasks involving isoluminant and luminant stimulation when sensitivity differences are taken into account.^73^^–^^76^

Although we did not explicitly examine the consequences of overlaps between color and size selectivity seen in Fig. 10, we did examine the separability of orientation and color selectivity in an experiment in which isoorientation regions were visible in V4 and V2. Consistent with the relative segregation of color and orientation processing in V2, we found that the addition of chromatic modulation did not dramatically alter the spatial distribution of orientation selectivity seen with achromatic stimulation and that there was relatively little orientation selectivity in the isoluminant plane (Fig. 13). This is consistent with a previous electrophysiological survey in which orientation tuning was independent of chromatic modulation in V2.^51^ It is also consistent with our imaging data which suggests that the majority of orientation selective cells in V2 respond to both achromatic and isoluminant stimuli. This is in sharp contrast to area V4, where there was a large region of orientation selectivity for isoluminant stimuli, and clear shifts in domains of orientation selectivity with changes in chromatic modulation. The inseparability of color and orientation tuning in V4 has been further observed in electrophysiological studies,^66^ where preferred direction in color space often depended on stimulus orientation, unlike in V2. Thus the overlap in V4 between functional domains that are largely segregated in V1 and V2, may yield a higher-order composite functional organization within V4.

A lack of clear segregation between color and orientation selectivity is not consistent with the conclusions reached in a previous optical imaging study of V2 and V4.^37^ In that study, in both V2 and V4, approximately 6% of pixels exhibited both orientation and color selectivity. By contrast we found that 70% in V2, and 95% in V4, of color selective pixels had orientation selectivity. The strict segregation of color and orientation suggested by the Tanigawa study^37^ is inconsistent with our electrophysiological measurements, and well as previous electrophysiological studies in V2.^23^^,^^24^^,^^77^ The difference is not readily explainable by differences in what portions of V4 were imaged. For example, when parafoveal stimulation is used, orientation organization in V4 is less strong,^36^ and the relative absence of strong orientation selectivity might lead to an apparent segregation. However, the Tanigawa study found little overlap between color and orientation domains even when foveal stimulation was used and a regular pattern of iso-orientation domains was visible. We believe the most likely explanation is that the spatial extent of color-specific responses was underestimated, and therefore, the chance of overlap between orientation selectivity and color selectivity reduced. This could happen for two reasons in the Tanigawa study. First, a single opponent color combination was used to characterize color regions, and second, stimuli potentially larger than the RF size, and therefore evoking surround suppression, were employed. Evidence in support of this conjecture in found in the supplementary data of Tanigawa^37^ which shows that when a broader range of colors and smaller stimuli are employed, larger overlaps are observed.

### 4.3 Higher-Order Functional Organization

When looking at the selectivities for any single property such as the incidence of color selectivity or orientation bandwidths, dramatic differences are often not visible between the early cortical areas. Our data is consistent with this: both V2 and V4 contain distinct regions of size, orientation, and color preference. The relative amount of activation seen with color-specific stimuli is similar between V2 and V4. Furthermore the dimensions and arrangement of color-selective regions are similar in the two areas. Instead, the most discernible difference between these visual areas, analogous to the differences seen between V1 and V2,^77^ is the increasing degree of association and interaction between different selectivities. Previous single unit studies are consistent with suggestion that higher levels of visual processing are associated with the progressive overlap of previously segregated functional streams. For example, the increase in receptive field size and reduction of strict retinotopy in higher visual areas can also be thought of as an association of previously segregated streams; in this case streams associated with different retinal locations. Other examples of functional desegregation and the formation of new patterns of functional organization appear throughout early visual cortex. In V1, magnocellular and parvocellular inputs as well as opponent color signals are combined. In V2, we see an increase in the overlap of cortical generated selectivities such as orientation and color. Finally, in V4 we see extensive overlap among color, size, and form, and the existence of a composite functional organization for a higher-order property, namely a combination of color and orientation that is not present in earlier areas.

Most importantly, our results demonstrate that these combinations are not just occurring at the single cell level, but rather can be observed among groups of neurons on a columnar scale. This need not be true: it is possible that the progressive integration of previously segregated signals might completely obscure any signs of functional organization. To borrow an example from the most studied case of cortical functional organization, the colinear summation of circularly symmetric geniculate receptive fields to form oriented cortical receptive fields does not necessarily imply the presence of columnar organization with respect to orientation in cortex. Although the exact genesis of this functional organization has not been definitively established, a number of models have been proposed in which organization arises on the basis of activity-based correlation rules and laminar and intracortical connectivity patterns. Because many of these physiological parameters, such as the size of intracortical patches and the laminar patterns of interareal projections are consistent between different cortical areas, functional organization may be a fundamental feature of cerebral cortex. Our finding that higher-order properties are functionally organized in V4 suggests that functional organization may be a natural result of the progressive convergence of functional streams and intrinsic anatomical and physiological tendencies toward functional clustering that exist throughout cortex.
